# Laryngology

**Published:** 1895-12

**Authors:** Henry J. Mulford

**Affiliations:** Buffalo, N. Y., Clinical instructor in diseases of the nose and throat at the Medical Department of Buffalo University


### Progress in Medical Science.

### LARYNGOLOGY.

Reported by HENRY J. MULFORD, M. D., Buffalo, N. Y.,
Clinical instructor in diseases of the nose and throat at the Medical Department of Buffalo University.

### The Surgical Treatment of Laryngeal Tuberculosis.

Abstract of papers read, with discussion, at the seventh annual summer meeting of the British Laryngological, Rhinological and Otological Association, 1895.

(*Journal Laryngology, Rhinology and Otology.*)

PROF. H. KRAUSE, Berlin, thinks the surgical treatment of laryngeal tuberculosis will be resolved favorably. It is as reasonable to remove tubercular tissue from the larynx as it is to remove it from any other situation. All cases may be helped. It is our duty to give ease to a patient by any means in our power. He quotes a case : " A woman in advanced pulmonary and laryngeal phthisis ; diffused infiltration and ulcerative destruction of almost entire laryngeal membrane ; enormous swelling of ary-epiglottic and pharyngo-epiglottic folds made swallowing impossible. Considerable pieces were cut from both folds, enabling her to swallow. Up to the time of her death there was no more trouble from the larynx."

In operating, it is necessary to remove all diseased tissue. No part of the larynx need be avoided. As a rule, the tissue cicatrises well. The healing process is hastened by application of lactic acid after operating. Patients bear the operation well. Many cases quickly relapse, but many cures have been made, lasting till death from the pulmonary condition.

Dr. THEODOR HERYNG : (1) Tubercle of the larynx can heal by itself without local treatment. (2) The chief indications in treatment are hygienic, dietetic and climatic. (3) The first important indication is removal of dysphagia. (4) The second is symptoms of stenosis. (5) The third relates to recovery of voice. (6) The healing of deep ulcers of the larynx is effected quickest by scraping or removal of tubercular tissue. (7) Surgical measures are indicated in most cases of recent origin ; (8) contraindicated in advanced cases. (9) Under cocaine the operation is painless. (10) One to 2 per cent. solution of pyoktanin is valuable in preventing inflammation after operation, applied twice daily. (11) Recurrence is frequent. (12) Nearly the whole of the upper larynx is accessible. (13) Operation does not affect a radical cure. (14) Success depends upon the character of the local affection, the patient, condition of lungs, thoroughness of operation, skill of operator and careful after-treatment. (15) Prognosis is difficult. (16) Serious bleeding rare. (17) Nonsuccess because treated too late. (18) This treatment demands the closest attention to detail, careful attention to after-treatment and observation over a long period. (19) Bad cases should be treated in climatic establishments. (20) The power of absorption in severe infiltrations, also the likelihood of healing of extensive ulcerations of larynx, with restoration of voice, has been proved to exist by anatomical and microscopic preparations as well as by clinical observation.

Dr. J. W. GLEITSMANN, New York : Curettement of the larynx has not received the attention in the United States that it has in Europe. Two reasons for this : the measure is thought too harsh by some; others think the small percentage of cures does not prove its usefulness. Curettement an important measure; should be used only on selected cases. It is not claimed that curettement will cure a concomitant pulmonary complication, nor can relapses be prevented. But if curettement improves or cures the laryngeal lesion the patient will be in better condition to fight other complications. Reports results in twelve cases: three dead; five, no improvement; four are without recurrence from six to ten months. Mentions one case cured by him seven years ago, by using curette, galvano-cautery and lactic acid. Had primary tuberculosis of pharynx and larynx; after five months' energetic treatment the ulcerations healed. Except slight relapse the following winter, patient is well today.

Mr. Lennox Browne mentions a case operated on by him in 1887, still living. There was such pain in swallowing, patient said she would sooner die by starvation than swallow. Extensive faucial and laryngeal ulceration. Under curetting and lactic acid complete recovery; lungs improved too. Has found so much benefit from curettement and lactic acid that he endorses the treatment. He thinks lactic acid is of no service without first curetting.

Dr. Luc has had an experience of six years with this treatment. Cure is rare, but relief follows in nearly every case. The greatest benefit is in those cases having great swelling of arytenoid region. Has had two cases of obstinate hemorrhage after cutting the epiglottis. As a local application has used phenol sulphuricinate exclusively with good results.

Dr. Law: Good climatic conditions necessary to success. Hard to find a suitable resort where special treatment may be had. Mentions a case whose larynx he curetted; after-treatment, lactic acid; worse at end of ten days. Sent her to the Riviera, after instructing patient how to apply the lactic acid to her own larynx. Ulcer perfectly healed after eight weeks.

Dr. Delavan, New York, came to Berlin doubting the value of surgical procedures in tuberculosis of the larynx. After seeing Professor Krause operate has a different idea as to its value. Is certain it is a valuable measure. Success depends upon the technique being observed with refined skill and with a high degree of precision, accuracy and care.

Dr. Heryng: Edema present disappears rapidly after operation. Some patients are so sensitive to laryngeal interference that to use a weak solution of lactic acid is to cause edema of infiltrated parts. As a rule, wounds made by the curette heal readily when the edges are not lacerated; they scarcely give rise to acute inflammation of the parts. To avoid infection of the wound paint with a 1 to 2 per cent. solution of pyoktanin.

Dr. MACKENZIE, Baltimore, thinks it established that laryngeal tuberculosis can be cured by intralaryngeal surgery, even if there be tuberculosis at other points. Does not advise operation where there may be very diffuse infiltration with ulceration. External operations of little value. Not justifiable until intralaryngeal methods fail; nor is the latter justifiable where there is very extensive disease of the larynx with grave lesions of the lungs or extensive tuberculosis elsewhere. Such patients die in spite of all treatment.

Prof. KRAUSE, in closing, said: I have particularly stated in my paper that I do not claim to cure these cases, but to relieve them. To relieve our patients is, according to my mind, one of the greatest works of our art. I do not recommend the treatment for general use, but we *must* do our duty in relieving the patient.

---

### EUROPEAN OPINION REGARDING THE USE OF ANTITOXIN IN DIPHTHERIA.

ON THE results of Heilserum therapy (discussion before the Thirteenth Congress for Internal Medicine, in Berlin): HEUBNER, Berlin: Of 1,332 cases treated without serum, 38.3 per cent. died; of 1,390 with serum, 19.1 per cent. died. A difference in the clinical process under serum cannot be determined. In 19 per cent. an exanthema followed injection, combined sometimes with fever and pain in joints. He favors serum treatment. BAGINSKY, Berlin: Mortality in Kaiser Friedrich's Hospital in years 1890 to 1894, 41 per cent.; of 525 cases treated with serum it was 15.81 per cent. Mortality of tracheotomies in other years, 59 per cent.; now, 38 per cent. Heart affections not influenced by serum treatment. Considers serum treatment the best and that the concomitant effects are of no moment. RAUKE, Munich: Mortality in the last year at the Münchener Hospital was 57 per cent.; during antitoxin period, 21 per cent. Of 96 cases of primary diphtheria, 63 had laryngo-stenotic symptoms; in 33 per cent. the stenosis disappeared after injection. In other epidemics it disappeared in only 5 per cent. Of intubated cases, 30.9 per cent. died; in other epidemics, 69 to 73 per cent. Exanthemata are without significance, considering the great effects of the treatment. SEITZ, Munich, had studied the complications observed before the use of serum ; thinks the kidney complications are not more frequent under serum. Exanthemata more frequent, but of no significance. STURYNIG, Jena, reports 59 cases treated with serum ; 20 per cent. died. WIEDERHOFER, Vienna, recommends antitoxin emphatically. TREUPPEL, Freiburg-i-Br., reports experiments on animals, which prove antitoxin produces, at times, transitory albuminuria, but that it has no harmful effects on the organism. SIEGERT, Strasburg, found albuminuria in 14 per cent. ; in cases treated with antitoxin, 41 per cent. VIERORDT, Heidelberg : Nature of the disease different in the various epidemics, and that, therefore, it is difficult to form a certain judgment on the effects of treatment. HEUBNER concludes that all authors have established that serum can be applied without any damage, that the mortality is diminished, and that, therefore, further application of it is indicated.

Therapeutics of diphtheria, with special reference to antitoxin. (Discussion before the British Laryngo- Rhino- and Otological Association; seventh annual summer meeting, July, 1895.) Dr. G. SIMS WOODHEAD had had a large laboratory experience with antitoxin, and has examined nearly 5,000 specimens, from over 2,000 cases of diphtheria, during the past eight or nine months. Has full confidence in the future of antitoxin. Very valuable in early stages ; less so in later stages when nerve and muscle lesions have occurred ; not good in mixed cases where other poisons are present with the toxins of diphtheria. Is convinced that the serum is harmless even when injected in large doses. No hesitation in advising prophylactic injection. Mr. LENNOX BROWNE : The preparation of antitoxin should be carefully selected, as specimens from different makers vary. Skin eruptions and joint pains are certainly septic. In 100 cases observed by him, 38 per cent. had these complications, while Moizard, of Paris, had only 14 per cent. Finds no improvement over classical remedies in using antitoxin. Mortality in one London hospital so great under antitoxin that it is no longer used. There is a tendency under serum treatment to increased kidney pressure. General condition of patient is not improved. Has been impressed by the great anemia persisting in children. Thinks there is some good in the treatment ; believes that injections are warranted in diseases that have resisted classical methods. We are not yet justified in accepting this treatment as specific in diphtheria. Statistics not reliable, because published too hastily. Deaths having occurred under prophylactic injection, we are not justified in using serum as a preventative. Dr. J. MACINTYRE thinks the general opinion is against serum being a specific. If it be true that in the laboratory immunity is thought possible, he hopes that improved methods in preparing and in using serum may make it of greater value. Dr. DE ROALDES: The abandonment of serum by one London hospital is fit for serious thought. He calls attention to report of a medical commission, in New Orleans, appointed to test antitoxin. There was no selection of patients; injected at any stage, under ordinary hygienic conditions. Death-rate did not exceed 8 per cent. in 250 cases. Its use cut the prevailing mortality one-third. Considers antitoxin of great value. Thinks it has come to stay. Mr. DALY is hopeful as to the future of antitoxin, but thinks we cannot afford to burn our bridges behind us in regard to classical methods of treatment. Has used antitoxin, but does not depend on it exclusively.

Dr. WOODHEAD, closing discussion: In taking statistics, it is not fair to any method of treatment to take a few selected cases. The serum treatment does not supply everything desired to overcome diphtheria. Anything in the old method, of service, must be used with the antitoxin. Antitoxin is not a panacea for all cases. In regard to renal changes, in *postmortems* on cases before the antitoxin treatment, a condition of nephritis was always found. This is due to the kidneys attempting to excrete the toxins. It may be pointed out that when albuminuria occurs as a result of the action of toxins on the kidneys that, if the antitoxin is pushed, the albuminuria is lessened; the toxins being so antagonised that they lose their stimulating power on the kidneys. This is now so well recognised that antitoxin is given in large doses to diminish albuminuria.

Discussion on serum treatment (Königlicher Verein der Aerzte in Budapest, March 2, 1895): HOGYES believes that the mortality in diphtheria is so variable that up to now statistics prove that Heilserum has no influence. In experiments upon animals the minimising effect is always applied; in men the curative effect is specially urged. WEISS recommends serum treatment. TAUGL thinks if serum had any effect the subsequent paralyses and nephritis would not occur. PERTIK also believes that statistics do not prove the efficacy of serum; further experiments should be performed.

PURJESZ thinks that the favorable statistics are obtained from the circumstance that now all the milder forms of diphtheria also come to the hospitals for treatment. He has observed one case of recurrence, first attack cured by serum. Three weeks later recurrence, serum given on first day of disease ; but result was death.

